# Adaptation Characteristics in the Range of Motion of the Shoulder Among Young Male Volleyball Players

**DOI:** 10.3390/jfmk10010067

**Published:** 2025-02-15

**Authors:** Kun-Yu Chou, Wan-Ling Wu, Chun-Wen Chiu, Shih-Chung Cheng, Hsiao-Yun Chang

**Affiliations:** 1 Graduate Institute of Athletics and Coaching Science, National Taiwan Sport University, Taoyuan 333325, Taiwan; chouhawk@gmail.com (K.-Y.C.); cjw19800310@gmail.com (C.-W.C.); shihchung@ntsu.edu.tw (S.-C.C.); 2 Department of Physical Education and Sport Sciences, National Taiwan Normal University, Taipei 106308, Taiwan; panther3336@gmail.com; 3 Department of Athletic Training and Health, National Taiwan Sport University, Taoyuan 333325, Taiwan

**Keywords:** overhead sports, flexibility, shoulder injury, glenohumeral internal rotation deficit

## Abstract

**Background/Objectives**: Repeated spiking and serving movements in volleyball can lead to alterations in shoulder range of motion among athletes, potentially increasing the risk of shoulder instability and injury. Hence, assessing and understanding the shoulder range of motion of volleyball players is a critical concern. Therefore, this study aimed to understand and evaluate the bilateral shoulder joint range of motion (ROM) in high-school male volleyball athletes and to discover the adaptation characteristics. **Methods**: Forty high-school male volleyball athletes participated in this study. Shoulder ROM measurements were taken via video with an iPhone 12 Pro Max, and we analyzed the ROM data using Kinovea software (Version 0.9.5) for both the dominant and non-dominant side. The shoulder ROM measurements included shoulder hyper-extension (SE), flexion (SF), internal rotation (IR), external rotation (ER), horizontal adduction (Sadd), and horizontal abduction (Sabd). After taking shoulder ROM measurements, the total rotational range of motion (TROM) was calculated based on the participants’ shoulder internal rotation and external rotation data, and we calculated the incidence of glenohumeral internal rotation deficiency (GIRD) among participants. Paired samples t-tests were used to analyze shoulder ROM differences between the dominant and non-dominant side. **Results**: The dominant side of the shoulder showed significantly lower internal rotation (dominant side: 42.17 ± 11.23°; non-dominant side: 52.14 ± 10.46°; *p* = 0.000) and total rotational ROM (dominant side: 137.11 ± 13.09°; non-dominant side: 141.96 ± 13.22°; *p* = 0.021) compared to the non-dominant side. Conversely, the dominant side of the shoulder exhibited significantly greater external rotation (dominant side: 94.96 ± 10.02°; non-dominant side: 89.83 ± 7.84°; *p* = 0.001) and shoulder horizontal adduction (dominant side: 44.87 ± 8.10°; non-dominant side: 39.60 ± 7.24°; *p* = 0.000) than the non-dominant side. No significant differences were found in other measured parameters. The incidence of glenohumeral internal rotation deficiency (GIRD) among all subjects was 37.5%. **Conclusions**: High-school male volleyball athletes in this study exhibited tightness in the posterior shoulder of their dominant side, indicating specific adaptations in shoulder ROM and a considerable prevalence of GIRD, observed in approximately one-quarter of the athletes. In conclusion, these data suggest that stretching and eccentric muscle training focusing on the posterior shoulder have potential value in mitigating these adaptations and reducing the risk of shoulder injuries.

## 1. Introduction

Volleyball is an overhead sport with high-frequency usage of spiking and serving movements. Volleyball players are expected to maximize the external rotation angle of their shoulder to prolong the acceleration distance of their spikes or serves, resulting in greater speed and power [1]. During an attack, the arm swing speed of volleyball players may reach 100 km/h [2]. In such a movement pattern, the key factor in maintaining the glenohumeral head stabilizer inside the joint fossa heavily relies on the surrounding soft tissues and muscles in charge of movement control [3].

The fast arm swing speed and the frequent repetition of spiking and serving movements make volleyball players prone to shoulder injuries [4]. Obana and associates [4] reported that shoulder injuries account for 12.2% of volleyball-related injuries; another study indicated that the prevalence of shoulder injuries among volleyball players was up to 24% [5]. These findings have shown us the significant risk of shoulder injuries among volleyball players, which is a non-negligible issue. Additionally, the musculoskeletal system of adolescents is still in its growing phase, and the development of the upper limb musculoskeletal system is usually completed at around 22–25 years of age [6,7]. The extensive training involving overhead movements can further increase the risk of shoulder injuries. Previous research has shown that high-intensity training in adolescent athletes may lead to abnormalities in shoulder mobility, skeletal growth, and muscle/tendon pathological changes in the upper limbs [8,9]. These abnormalities can result in shoulder tendinitis and scapular dyskinesis [8,9]. Frisch and associates [10] found that 40% of high-school female volleyball players experienced non-traumatic shoulder pain, but only 33% took rest. To mitigate shoulder injury issues, it is essential not only to regulate the intensity and volume of training but also to address the intrinsic risk factors, such as range of motion (ROM), associated with young volleyball players.

Glenohumeral internal rotation deficiency (GIRD) is a common shoulder ROM limitation problem among overhead athletes. It is also one of the intrinsic risk factors for shoulder injuries [11,12]. According to Manske et al. [12], glenohumeral internal rotation deficiency (GIRD) is defined as a reduction in internal rotation exceeding 10 degrees, accompanied by a reduction in total rotational motion (TROM) of the dominant shoulder exceeding 5 degrees. GIRD occurs in overhead athletes due to the eccentric contraction of soft tissues in the posterior shoulder during the deceleration phase of the arm swing, leading to microtrauma and subsequent tightness of these tissues [11,13,14]. The study by Mizoguchi and associates [15] showed that 38% of young volleyball players experienced a reduction in the internal rotation range of motion (ROM) and total rotation range of motion (TROM) of the dominant shoulder. Previous studies have indicated that posterior shoulder tightness may also lead to a decrease in shoulder horizontal adduction and internal rotation ROM, as well as an increase in external rotation ROM, which may all be contributing to the development of GIRD [16,17]. Schmalzl and associates [18] found that in adult volleyball players, GIRD of less than 10 degrees was significantly associated with shoulder impingement and decreased TROM. Alqarni and associates [19] reported that players with a history of shoulder pain had larger ROM differences in GIRD and TROM than those without a history of shoulder pain. These findings suggest that a reduction in TROM often accompanies GIRD and is significantly correlated with shoulder pain. Hence, understanding these adaptive changes is of utmost importance for preventing and treating shoulder injuries in youth volleyball players. Therefore, this study aimed to investigate and evaluate bilateral shoulder range of motion (ROM), quantify the prevalence of glenohumeral internal rotation deficiency (GIRD), and compare shoulder ROM between athletes with and without GIRD among male high-school volleyball players.

## 2. Materials and Methods

### 2.1. Study Design and Participants

This study is a prospective cross-sectional study. The sample size was calculated using G*Power software (G*Power 3.1.9.6 for Windows, Heinrich-Heine-Universität Düsseldorf, Düsseldorf, Germany), with the effect size set at 0.5, the significance level (α) at 0.05, and the statistical power at 0.8. The minimum required sample size was determined to be 34 participants, and a total of 40 participants were recruited for this study.

During the preseason phase of the 2023 season, data on bilateral shoulders were collected from Taiwanese high-school volleyball players. The participants in this study were recruited players from the top four teams in the ranking of the Taiwan High School Volleyball League (HVL).

A total of 40 high-school male volleyball athletes participated in the study. Among the participants, 34 were right-handed and 6 were left-handed. The participants’ average age was 17.7 ± 0.9 years, their average height was 180.0 ± 5.9 cm, their average weight was 71.1 ± 7.5 kg, and their average volleyball experience was 6.5 ± 2.6 years. The inclusion criteria were as follows: no history of upper limb injury, fracture, dislocation, subluxation, or any neurological disorders in the past year; negative results on special shoulder tests administered by a physical therapist; and a minimum training load of four days per week in addition to at least three years of specialized training in volleyball. All participants were informed about the experimental procedures, and both participants and their parents signed the informed consent forms. The study protocol adhered to the Declaration of Helsinki and was approved by the Human Research Ethics Committee of Fu Jen Catholic University in Taiwan (IRB No: FJU-IRB C110139).

### 2.2. Procedure

This study was conducted with an iPhone 12 Pro Max (Apple Inc., Cupertino, CA, USA), recording videos (1080 HD/60 fps) of the active ROM of the shoulder. The videos were then analyzed using Kinovea software (Version 0.9.5; Kinovea open source project, www.kinovea.org, access data: 18 July 2023) to measure shoulder ROM. Kinovea software is a free 2D motion analysis software and low-cost technology which has been previously used in sports sciences and clinical and research fields. Kinovea software also has good validity in terms of achieving the right angle [20].

**Figure 1:**
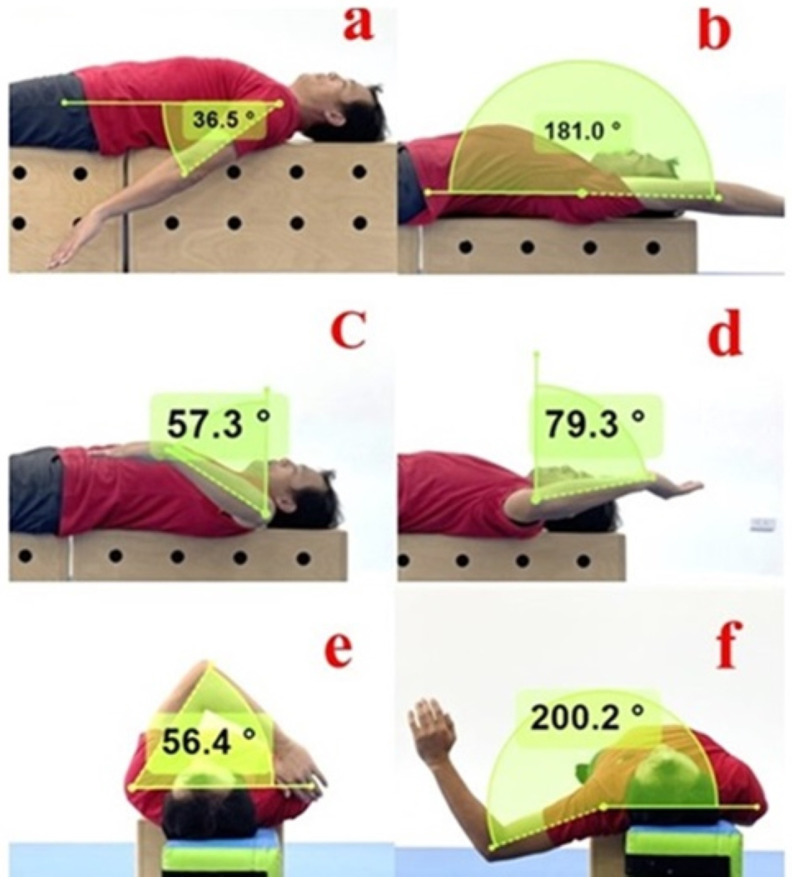
Shoulder range of motion measurement. (**a**) Shoulder hyper-extension; (**b**) shoulder flexion; (**c**) shoulder internal rotation; (**d**) shoulder external rotation; (**e**) shoulder horizontal adduction; (**f**) shoulder horizontal abduction.

The shoulder ROM measurements included shoulder hyper-extension (SE), flexion (SF), internal rotation (IR), external rotation (ER), horizontal adduction (Sadd), and horizontal abduction (Sabd) (Figure 1). All measurements underwent test–retest reliability assessments before the formal pilot testing, with reliability coefficients ranging from 0.984 to 0.998 (SE: 0.984; SF: 0.993; IR: 0.997; ER: 0.991; TROM: 0.993; Sadd: 0.998; and Sabd: 0.995). The dominant and non-dominant sides were tested randomly. In this study, the dominant hand was defined as the upper limb used by the volleyball player for attacking movements. All measurements and data collection were performed by one researcher; each measuring item was tested twice and the average value was used for further statistical analysis. All tests were conducted at each high-school gym. Participants performed a 15 min warm-up, including jogging and stretching. After 10 min of warming up, participants drew lots to determine the order of testing and then had their shoulder ROM measured in sequence. There was a 5 min rest period between each measurement item. Below is the procedure of each shoulder ROM measurement.

Shoulder Hyper-Extension Measurement (Figure 1a): The participant lay supine on a platform with their arm positioned alongside their body. The researcher manually stabilized their scapula. The participant then moved their arm posteriorly to its limit. Upon reaching maximal extension, the video recording was stopped. The video was then analyzed using Kinovea to measure the joint angle. The shoulder joint center was used as the axis to draw two straight lines: one parallel to the lateral midline of the trunk and the other parallel to the midline of the humerus. The angle between these two lines was recorded as the shoulder hyper-extension angle [21].

Shoulder Flexion Measurement (Figure 1b): The participant lay supine on a platform with their arm positioned alongside their body. The researcher manually stabilized their scapula. The participant then raised their arm overhead into flexion to the maximal possible degree. Upon reaching maximal flexion, the video recording was stopped. The video was then analyzed using Kinovea to measure the joint angle. The shoulder joint center was used as the axis to draw two lines: one parallel to the lateral midline of the trunk and the other parallel to the midline of the humerus. The angle between these two lines was recorded as the shoulder flexion angle [21].

Shoulder Internal/External Rotation Measurement (Figure 1c,d): The participant lay supine on a platform with their shoulder abducted to 90 degrees, their elbow flexed to 90 degrees, and their forearm positioned with their palm facing down. For internal rotation, the participant moved their arm toward their feet till their end range. Upon reaching maximal internal rotation, the video recording was stopped. The video was then analyzed using Kinovea to measure the joint angle. Olecranon was used as the axis, and two lines were drawn: one perpendicular to the ground and the other parallel to the longitudinal axis of the ulna. The angle between these two lines was recorded as the shoulder internal rotation angle [20]. For external rotation, the participant’s forearm was again positioned with their palm facing down, and the participant moved their arm toward their head to the maximal limit. Upon reaching maximal external rotation, the video recording was stopped. The video was then analyzed using Kinovea to measure the joint angle. Using the olecranon as the axis, two lines were drawn: one perpendicular to the ground and the other parallel to the longitudinal axis of the ulna. The angle between these two lines was recorded as the shoulder external rotation angle [21].

Shoulder Horizontal Adduction Measurement (Figure 1d): The participant lay supine on a platform while the researcher manually stabilized their scapula. The participant then moved their arm horizontally across their chest toward their opposite shoulder to the maximal limit. Upon reaching maximal horizontal adduction, the video recording was stopped. The video was then analyzed using Kinovea to measure the joint angle. Using the acromion as the axis, two straight lines were drawn: one parallel to the top of the shoulder and the other parallel to the longitudinal axis of the humerus. The angle between these two lines was recorded as the shoulder horizontal adduction angle [22]. This measurement assesses the flexibility of the posterior shoulder; a larger angle indicates greater tightness in the posterior shoulder.

Shoulder Horizontal Abduction Measurement (Figure 1e): The participant lay supine on a platform with their shoulder abducted to 90 degrees. The participant then moved their arm horizontally and posteriorly to the maximal limit. Upon reaching maximal horizontal abduction, the video recording was stopped. The video was then analyzed using Kinovea to measure the joint angle. Using the acromion as the axis, two straight lines were drawn: one parallel to the top of the shoulder and the other parallel to the longitudinal axis of the humerus. The angle between these two lines was recorded as the shoulder horizontal abduction angle [22]. This measurement assesses the flexibility of the anterior shoulder, specifically the pectoralis muscle; a larger angle indicates greater anterior shoulder flexibility.

After completing the measurements, the total rotational range of motion (TROM) and glenohumeral internal rotation deficiency (GIRD) were calculated using the participants’ shoulder internal and external rotation data. The formula for TROM is as follows:

TROM = Shoulder Internal Rotation (IR) + Shoulder External Rotation (ER)

GIRD was determined using the following criteria: if the difference between the non-dominant side’s internal rotation and the dominant side’s internal rotation was greater than 10 degrees, and the difference between the non-dominant side’s TROM and the dominant side’s TROM was greater than 5 degrees, the participant was classified as having GIRD [15]. The occurrence of GIRD was calculated by dividing the number of GRIDs by the total number of people, and then multiplying this by 100%.

### 2.3. Statistical Analysis

The data were analyzed using SPSS 20.0 for Windows (IBM Corp., Armonk, NY, USA). Descriptive statistics, including the mean and standard deviation (SD), were presented. The Shapiro–Wilk test was conducted to assess the normality of the data. If the data of the participants exhibited a normal distribution, a paired-sample t-test was used to compare the differences in shoulder ROM between the dominant and non-dominant sides of the participants. Finally, Cohen’s d was calculated to determine the effect size, with 0.2 indicating a small effect, 0.5 a medium effect, and 0.8 a large effect. The significance level (α) was set at 0.05.

## 3. Results

This study compared shoulder ROM between the dominant and non-dominant sides of high-school male volleyball players, with the results shown in Table 1. The findings indicate that IR on the dominant side was significantly less than on the non-dominant side by 9.97 ± 10.25 degrees (*p* = 0.000, Cohen’s *d* = 0.92). TROM on the dominant side was significantly less than on the non-dominant side by 4.85 ± 12.72 degrees (*p* = 0.021, Cohen’s *d* = 0.37). ER on the dominant side was significantly greater than on the non-dominant side by 5.13 ± 9.1 degrees (*p* = 0.001, Cohen’s *d* = 0.57). Sadd on the dominant side was significantly greater than on the non-dominant side by 5.27 ± 6.50 degrees (*p* = 0.000, Cohen’s *d* = 0.69). The other parameters failed to reach statistical significance, including SE (*p* = 0.106, Cohen’s *d* = 0.24), SF (*p* = 0.368, Cohen’s *d* = 0.07), and Sabd (*p* = 0.417, Cohen’s *d* = 0.08).

Among the 40 participants, 15 met the criteria for GIRD, indicating that 37.5% of the subjects had GIRD. The shoulder ROM performance of those with GIRD is shown in Table 2. For the GIRD group, IR and TROM on the dominant side were significantly less than on the non-dominant side: IR was less by 18.68 ± 7.23 degrees (*p* = 0.000, Cohen’s *d* = 1.72), and TROM was less by 17.32 ± 10.25 degrees (*p* = 0.000, Cohen’s *d* = 1.37). Additionally, Sadd on the dominant side was significantly greater by 6.71 ± 6.87 degrees (*p* = 0.002, Cohen’s *d* = 0.88). The other parameters did not reach statistical significance, including SE (*p* = 0.817, Cohen’s *d* = 0.07), SF (*p* = 0.245, Cohen’s *d* = 0.17), ER (*p* = 0.543, Cohen’s *d* = 0.15), and Sabd (*p* = 0.656, Cohen’s *d* = 0.05).

## 4. Discussion

This study aimed to evaluate the shoulder ROM in male high-school volleyball players. The results show that the dominant side had significantly less internal rotation and total rotational ROM than the non-dominant side, while the dominant side had significantly greater external rotation and shoulder horizontal adduction. However, no significant difference was shown in shoulder flexion, hyper-extension, and horizontal abduction. Previous research indicates that overhead athletes often develop posterior shoulder tightness due to long-term overhead arm movements, leading to increased Sadd and decreased IR [16,17], which is consistent with our findings. These results might be due to shoulder adaptations caused by overuse, resulting in posterior shoulder tightness. Past research on volleyball players has paid little attention to Sadd and posterior shoulder tightness. However, our study observed that the Sadd of the dominant arm in high-school male volleyball players was significantly greater than that of the non-dominant arm. Vad and associates found a high correlation between reduced IR and shoulder injuries and pain [23]. This means that greater Sadd ROM and posterior shoulder tightness may be risk factors for shoulder injury for youth volleyball players. To prevent such conditions, stretching exercises targeting the posterior shoulder muscles for youth volleyball players to maintain appropriate shoulder ROM should be performed.

Additionally, research by Schmalzl et al. [18] on handball and volleyball players also reported similar results. Previous studies have also indicated that overhead athletes often experience decreased IR, accompanied by increased ER and reduced total rotational range of motion (TROM) [18,24,25,26], which aligns with our findings. However, Mizoguchi and associates [27] found that adolescent volleyball players had significantly increased ER and decreased IR, but no significant difference in TROM, partially aligning with our results. In volleyball, players ought to achieve maximum external rotation of the shoulder to enhance the power and speed of their attacks and serve [1]. Repeated volleyball attacks and serves can lead to increased ER. This may also cause pathological changes in the posterior shoulder. This condition can be managed by performing the sleeper stretch or cross-body stretches and strengthening the shoulder external rotators muscles, especially via eccentric exercises, as a preventive strategy to reduce the risk of shoulder injuries [25,26,27,28,29].

This study further investigated the incidence of GIRD among youth volleyball players, finding it to be 37.5%. Previous studies on adolescent volleyball players have reported GIRD incidence rates of 38.2% [30] and 38.5% [24], which are similar to our findings. Additionally, Schmalzl and associates [18] reported an even higher incidence rate of 72% in male adult handball and volleyball players. Past research has indicated that volleyball players with GIRD often experience increased ER and decreased TROM [24,29], which conforms with our results. However, Mizoguchi and associates [27] found that adolescent volleyball players with GIRD exhibited significant decreases in both ER and TROM, partially matching up with our findings. Previous studies have suggested that increased ER in the dominant hand is a specific adaptation in overhead athletes, allowing for better athletic performance [24,29]. The difference between adult and adolescent players may come from the accumulation of tissue micro-damage caused by long-term training and repeated overhead movements in adults. Research has indicated that athletes with GIRD who continue to train may experience shoulder pain and an increased risk of injury [24,25,26,27,28,29,30,31], leading to absence from practices and competitions. However, this situation has also been found in the youth players in this study. This means that changes and adaptations in shoulder ROM need to be monitored, starting with youth players.

This study did not find significant differences in SE, SF, and Sabd. Previous research on the shoulder ROM of youth volleyball players has not specifically tested SE and SF, making it difficult to perform a comparison. However, when the participants were categorized into GIRD and non-GIRD subgroups, notable differences were observed. In the non-GIRD group, the SE and IR ROM of the dominant arm were significantly less than those of the non-dominant arm. Conversely, the ER and Sadd ROM of the dominant arm were significantly greater than those of the non-dominant arm. These findings suggest that athletes classified as non-GIRD may already exhibit early symptoms of GIRD and increased bicep tightness. Therefore, it is essential to also monitor and address the flexibility of the non-GIRD group to prevent the progression of shoulder dysfunction. In addition, a previous study has indicated that a decrease in Sabd is associated with lower back pain [15]. Although no differences were found in SF and Sabd parameters, they still fall within the scope of shoulder mobility and should be closely monitored.

Overall, research on shoulder ROM and GIRD in youth volleyball players is relatively scarce. Our study found that youth volleyball players exhibit specific adaptations in their dominant arm, particularly in IR, ER, TROM, and Sadd. In addition, the prevalence of GIRD is notably high in this population. Future research should aim to conduct long-term follow-up studies on this group and span adolescent, young, and adult players to understand how shoulder ROM changes. Our study recommended that youth volleyball players engage in eccentric exercises for posterior shoulder muscles and specific stretches that enhance shoulder mobility and strength.

This study has several limitations that need to be addressed. One of the limitations of this study is that it only collected data from players who participated in the competition, excluding players who did not participate in the competition. These players who did not participate in the competition may have an affected performance due to injuries or insufficient shoulder mobility, so they were unable to participate in the competition. However, our study did not include these players. Second, our participants came from four high schools. The variance in training schedules and volume among the teams could have led to different levels of muscle fatigue [32,33], and fatigue might affect shoulder strength, proprioception, and range of motion, representing possible risk factors for overuse shoulder injury [32,33]. We did not record related information on training volume or intensity. Third, we did not categorize the subgroups based on the volleyball positions of the participants. This may affect differences in shoulder ROM due to different playing positions. Future research should consider tracking the training volume and intensity of players through different competition cycles to understand the fatigue effect on shoulder strength and to better understand the occurrence of specific shoulder ROM adaptations in young male volleyball players.

## 5. Conclusions

The high-school male volleyball athletes in this study exhibited tightness in the posterior shoulder of their dominant side, indicating specific adaptations in shoulder ROM and a considerable prevalence of GIRD, observed in approximately one-quarter of the athletes. In conclusion, these data suggest that stretching and eccentric muscle training focusing on the posterior shoulder have potential value in mitigating these adaptations and reducing the risk of shoulder injuries.

## Figures and Tables

**Table 1:** Shoulder range of motion results.

ROM	Dominant Side	Non-Dominant Side	Difference Between Bilateral Sides ^#^	*p*	Cohen’s *d*
	Mean ± SD	Mean ± SD	Mean ± SD		
SE	51.43 ± 4.60	52.74 ± 6.44	−1.31 ± 5.03	0.106	0.24
SF	177.55 ± 9.00	178.17 ± 8.04	−0.62 ± 4.32	0.368	0.07
IR	42.17 ± 11.23	52.14 ± 10.46	−9.97 ± 10.25	0.000 *	0.92
ER	94.96 ± 10.02	89.83 ± 7.84	5.13 ± 9.10	0.001 *	0.57
TROM	137.11 ± 13.09	141.96 ± 13.22	−4.85 ± 12.72	0.021 *	0.37
Sadd	44.87 ± 8.10	39.60 ± 7.24	5.27 ± 6.50	0.000 *	0.69
Sabd	216.46 ± 10.08	215.52 ± 11.38	0.94 ± 7.15	0.417	0.08

Note: Unit: degree; SE: shoulder hyper-extension; SF: shoulder flexion; IR: internal rotation; ER: external rotation; TROM: total rotational range of motion; Sadd: shoulder horizontal adduction; Sabd: shoulder horizontal abduction; * *p* < 0.05; Cohen’s *d* ≤ 2 means low effect; 2 < Cohen’s *d* ≤ 5 means mild effect; Cohen’s *d* > 5 means high effect. ^#^ The difference between the bilateral sides means the ROM of the dominant side minus non-dominant side.

**Table 2:** GIRD athletes’ shoulder range of motion results.

ROM		Non-GIRD (N = 25)			GIRD (N = 15)		
		Mean ± SD	*p*	Cohen’s *d*	Mean ± SD	*p*	Cohen’s *d*
SE	D	52.25 ± 4.75	0.035 *	0.35	50.05 ± 4.14	0.817	0.07
	ND	54.13 ± 5.51			50.43 ± 7.37		
SF	D	177.11 ± 9.53	0.726	0.01	178.29 ± 8.30	0.245	0.17
	ND	177.25 ± 7.90			179.71 ±8.31		
IR	D	44.69 ± 10.87	0.007 *	0.47	37.95 ± 10.87	0.000 *	1.72
	ND	49.45 ± 9.54			56.63 ± 10.68		
ER	D	96.21 ± 9.46	0.000 *	0.84	92.87 ± 10.89	0.543	0.15
	ND	88.83 ± 8.10			91.51 ± 7.35		
TROM	D	140.88 ± 11.81	0.056	0.21	130.81 ± 13.06	0.000 *	1.37
	ND	138.26 ± 12.66			148.13 ± 12.12		
Sadd	D	43.30 ± 8.05	0.002 *	0.58	47.49 ± 7.74	0.002 *	0.88
	ND	38.90 ± 7.07			40.78 ± 7.61		
Sabd	D	218.80 ± 8.59	0.257	0.20	212.71 ± 11.41	0.656	0.05
	ND	216.89 ± 10.07			213.33 ±13.29		

Note: Unit: degree; GIRD: glenohumeral internal rotation deficiency; SE: shoulder hyper-extension; SF: shoulder flexion; IR: internal rotation; ER: external rotation; TROM: total rotational range of motion; Sadd: shoulder horizontal adduction; Sabd: shoulder horizontal abduction; D: dominant side; ND: non-dominant side; * *p* < 0.05; Cohen’s *d* ≤ 2 means low effect; 2 < Cohen’s *d* ≤ 5 means mild effect; Cohen’s *d* > 5 means high effect.

## Data Availability

The data that support the findings of this study are available on request from the corresponding author. The data are not publicly available due to privacy or ethical restrictions.
